# Integrating TSPO-PET imaging with metabolomics for enhanced prognostic accuracy in multiple sclerosis

**DOI:** 10.1136/bmjno-2025-001026

**Published:** 2025-04-16

**Authors:** Daniel E Radford-Smith, Abi G Yates, Tereza Kacerova, Marjo Nylund, Marcus Sucksdorff, Markus Matilainen, Eline Willemse, Johanna Oechtering, Aleksandra Maleska Maceski, David Leppert, Jens Kuhle, Fay Probert, Daniel C Anthony, Laura Airas

**Affiliations:** 1 Department of Pharmacology, University of Oxford, Oxford, UK; 2 Department of Chemistry, University of Oxford, Oxford, UK; 3 Turku PET Centre, University of Turku, Turku University Hospital and Åbo Akademi University, Turku, Finland; 4 Clinical Neurosciences University of Turku, Turku, Finland; 5 Neurocenter, Turku University Hospital, Turku, Finland; 6 InFLAMES Research Flagship, University of Turku, Turku, Finland; 7 Multiple Sclerosis Centre, Departments of Biomedicine and Clinical Research, University Hospital and University of Basel, Basel, Switzerland; 8 Department of Neurology, University Hospital and University of Basel, Basel, Switzerland; 9 Research Center for Clinical Neuroimmunology and Neuroscience Basel, University Hospital and University of Basel, Basel, Switzerland; 10 Department of Clinical Research, University Hospital and University of Basel, Basel, Switzerland

**Keywords:** MULTIPLE SCLEROSIS, PET

## Abstract

**Background:**

Predicting disease progression in multiple sclerosis (MS) remains challenging. PET imaging with 18 kDa translocator protein (TSPO) radioligands can detect microglial and astrocyte activation beyond MRI-visible lesions, which has been shown to be highly predictive of disease progression. We previously demonstrated that nuclear magnetic resonance (NMR)-based metabolomics could accurately distinguish between relapsing-remitting (RRMS) and secondary progressive MS (SPMS). This study investigates whether combining TSPO imaging with metabolomics enhances predictive accuracy in a similar setting.

**Methods:**

Blood samples were collected from 87 MS patients undergoing PET imaging with the TSPO-binding radioligand ^11^C-PK11195 in Finland. Patient disability was assessed using the expanded disability status scale (EDSS) at baseline and 1 year later. Serum metabolomics was performed to identify biomarkers associated with TSPO binding and disease progression.

**Results:**

Greater TSPO availability in the normal-appearing white matter and perilesional regions correlated with higher EDSS. Serum metabolites glutamate (p=0.02), glutamine (p=0.006), and glucose (p=0.008), detected by NMR, effectively distinguished future progressors. These three metabolites alone predicted progression with the same accuracy as TSPO-PET imaging (AUC 0.78; p=0.0001), validated in an independent cohort. Combining serum metabolite data with PET imaging significantly improved predictive power, achieving an AUC of 0.98 (p<0.0001).

**Conclusion:**

Measuring three specific serum metabolites is as effective as TSPO imaging in predicting MS progression. However, integrating TSPO imaging with serum metabolite analysis substantially enhances predictive accuracy. Given the simplicity and affordability of NMR analysis, this approach could lead to more personalised, accessible treatment strategies and serve as a valuable tool for clinical trial stratification.

**WHAT IS ALREADY KNOWN ON THIS TOPIC**

- Predicting disease progression in multiple sclerosis (MS) is challenging due to disease heterogeneity and the lack of validated biomarkers. 18 kDa translocator protein (TSPO)-PET imaging detects microglial activation associated with progression, while nuclear magnetic resonance (NMR) metabolomics distinguishes MS subtypes. Despite the individual promise of these methods, no studies have yet combined them to enhance prognostic accuracy.

**WHAT THIS STUDY ADDS**

- The combination of three serum metabolites—glutamate, glutamine, and glucose—effectively predicted MS progression (AUC 0.78; p=0.0001), with accuracy comparable to TSPO-PET imaging. When integrated with TSPO-PET imaging, these biomarkers significantly enhanced predictive performance, achieving an AUC of 0.98 (p<0.0001).

**HOW THIS STUDY MIGHT AFFECT RESEARCH, PRACTICE OR POLICY**

- The integration of TSPO-PET imaging with serum metabolome analysis offers a powerful tool for personalising treatment strategies in MS by yielding physiologically relevant predictions that align with patient outcomes. Given the simplicity and affordability of NMR analysis, this approach has the potential to enhance patient management and enable more tailored therapies, ultimately advancing personalised medicine in MS.

## Introduction

 For individuals with multiple sclerosis (MS), there is a lack of clinical markers allowing prediction of disease progression to guide timely therapy selection. Increased binding of the 18 kDa translocator protein (TSPO) radioligand ^11^C-PK11195 has been shown to be predictive of progression at baseline.^1^,^3^ Patients whose expanded disability status scale (EDSS) score increased over a 4 year period exhibited stronger TSPO-PET signals at baseline, particularly in the normal-appearing white matter (NAWM) and perilesional areas.^3^ TSPO binding is also greater in secondary progressive (SPMS) compared with relapsing-remitting (RRMS) patients,^1^ suggesting a link between neuroinflammation activity and disease progression.^4^ TSPO is an outer mitochondrial membrane protein involved in cholesterol transport and control of mitochondrial activity.^3^ During CNS inflammation, TSPO expression increases in activated macrophages, microglia and astrocytes, and the toxic activities of the pro-inflammatory glial cells are thought to contribute to MS degenerative mechanisms.^5^ Additionally, increased TSPO availability reflects a heightened density of activated innate immune cells, underscoring the value of TSPO in monitoring ongoing neuroinflammatory processes.^6^ While TSPO-PET is highly effective in revealing chronic brain pathology in vivo, its broader clinical application faces feasibility challenges due to high technical demands and costs. Therefore, alternative strategies are being explored to identify patients with elevated brain-compartmentalised innate immune cell activity and to improve the prediction of MS progression.

Nuclear magnetic resonance (NMR) metabolomics has become a popular biomarker identification technique. Furthermore, recent studies indicate that the serum metabolome may more sensitively detect brain inflammation than other biomarkers measurable from blood.^7 8^ We have consistently demonstrated that serum NMR-based metabolomics enables differentiation between the stages of MS,^8^ and that metabolite biomarkers can discriminate between patients in relapse and remission.^7 8^

Here, we sought to determine if the serum metabolome could identify MS individuals exhibiting high ^11^C-PK11195 TSPO binding and, by extension, predict disease progression similarly to ^11^C-PK11195 TSPO binding. Given that NMR metabolomics is a simple, non-invasive and low-cost technique, it presents a potential alternative for monitoring disease progression in MS, which could reduce the need for more complex and expensive imaging methods like PET scans.

## Materials and methods

### Study subjects and procedures

To identify biomarkers of progression within a 1 year time frame, patients were classified as progressors with any confirmed positive change in EDSS score (ΔEDSS) ≥0.5, and as non-progressors with a ΔEDSS score ≤0. This stratification reflects a clinically significant change in disability, which can be indicative of disease worsening over a relatively short period of time. There were no significant differences between progressors and non-progressors in age, sex, disease duration or EDSS baseline scores, as verified by statistical analysis (table 1).

**Table 1:** Baseline characteristics of multiple sclerosis progressors and non-progressors (all results are presented as means and were compared using Fisher’s exact test for categorical variables and Wilcoxon test for continuous variables)

	Non-progressors (n=52)	Progressors (n=23)	P value
Age (mean years)	46.4	48.9	0.24
Sex (% female)	82.2	70.0	0.33
Race/ethnicity			>0.99
%Caucasian	100.0	100.0	
% Other	0.0	0.0	
Disease type			0.16
% RRMS	73.0	52.2	
% SPMS	13.5	30.4	
% Other/unknown	13.5	17.4	
Disease duration (mean years)	11.5	12.5	0.56
EDSS	2.8	3.6	0.08
Medication at sampling (%)			0.32
None	21.1	30.4	
Dimethyl fumarate	7.7	0.0	
Fingolimod	19.2	21.7	
Glatiramer acetate	5.8	13.0	
IFN-β	9.6	8.7	
Natalizumab	13.5	13	
Rituximab	1.9	8.7	
Teriflunomide	21.2	4.3	

EDSS, expanded disability status scale; RRMS, relapsing-remitting multiple scleoris; SPMS, secondary progressive multiple sclerosis.

#### Turku cohort

The Turku cohort MS patients were recruited from the outpatient clinic of the Division of Clinical Neurosciences at the University Hospital Turku, Finland, between 2009 and 2021. The cohort consisted of 87 Caucasian MS patients who underwent PET and MRI scans and a blood draw. For 75/87 (86%) patients, the EDSS score was assessed at the time of imaging and blood sampling and 1 year later (table 1, online supplemental eMethods 1).

#### Swiss MS cohort (SMSC; subset)

SMSC is a prospective multicentre cohort study performed across eight Swiss academic medical centres, conducted between 2012 and 2022. The validation cohort included 37 individuals with MS who underwent MRI scans and blood draw. The EDSS scores were evaluated at the baseline and 1 year later (online supplemental SI eTable 1,eMethods 2).^9^

#### Patient and public involvement

No patient and public involvement.

### Standard protocol approvals and patient consents

The Ethical Committee of the Hospital District of Southwest Finland approved the study, and written informed consent was obtained from all participants according to the Declaration of Helsinki. SMSC was approved by the ethics committees of all participating centres. Participants provided written informed consent. This study followed the Strengthening the Reporting of Observational Studies in Epidemiology reporting guidelines.

### MRI acquisition and data analysis

For the evaluation of MS pathology and for the acquisition of anatomic reference for the PET images, MRI with a Gyroscan Intera 1.5 T (n=13), 3 T Ingenuity TF PET/MR (n=74) scanners (Philips) was performed. For detailed MRI data acquisition protocol, refer to online supplemental eMethods 3, 4.

### Radioligand production and PET imaging acquisition

The radiochemical synthesis of ^11^C-PK11195 followed previously described methods (refer to online supplemental eMethods 5.^2^ The mean injected dose was 481±37 MBq (mean±SD). The median molar activity at injection was 55 (IQR 38–95) MBq/nmol. The molar activities were determined from the measured activity of the end product at the end of synthesis and the amount of substance based on the HPLC analysis of the end product. All molar activities are time-corrected to the injection time. PET scans were performed using a brain-dedicated ECAT HRRT scanner (CTI/Siemens) with a 2.5 mm spatial resolution. A 60 min dynamic PET scan began with an intravenous bolus injection of the ^11^C-PK11195 radioligand, following a 6 min transmission scan for attenuation correction using a ^137^Cs point source.

### PET post-processing and analysis

PET images were reconstructed as previously described using 17 time frames, coregistered to T1 MRI and resampled to match the MRI voxel size 1 mm × 1 mm × 1 mm. The specific binding of ^11^C-PK11195 was evaluated using distribution volume ratio (DVR) in pre-specified ROIs. For ^11^C-PK11195 DVR estimation, the time–activity curve for a reference region devoid of specific TSPO binding was acquired for each PET session using a supervised cluster algorithm with four predefined kinetic tissue classes (SuperPK software).^10 11^ For detailed PET processing information, refer to online supplemental eMethods 6.

### NMR sample preparation

Serum samples were thawed at room temperature and ultracentrifuged at 1 00 000 g for 30 min at 4°C, in line with previous studies.^8^ 100 µL of supernatant was combined with 450 µL of NMR buffer (75 mM sodium phosphate buffer prepared in D_2_O, pH 7.4) and stored at −80°C until analysis. On the day of analysis, samples were thawed at room temperature and transferred to 5 mm NMR tubes.

### ^1^H NMR metabolomics data acquisition and processing

All samples were measured using the 700-MHz Bruker AVII spectrometer operating at 16.4T, equipped with a ^1^H (^13^C/^15^N) TCI cryoprobe, as described previously.^8^ Samples were held at 310K, and spectra were acquired using a water suppression with a transverse relaxation filter that eliminates distortions (WASTED) sequence, as previously described.^12^

All spectra were processed in Topspin 4.0.7 (Bruker) and ACD/NMR processor academic edition 12.01 (Advanced Chemistry Development, Inc). The spectral regions were manually binned into ‘buckets’ corresponding to individual metabolites. Metabolite assignment was performed using literature reviews,^13^ the HMDB database^14^ and 2D total correlation spectroscopy experiments. For detailed information, refer to online supplemental eMethods 7, 8.

### Statistical analysis

For metabolomics data, the integrals of the scaled spectral buckets were imported into R software v4.2.1 (R foundation for statistical computing). The diagnostic potential of metabolites for disease progression was evaluated using receiver operator curves (ROC), area under the curve (AUC) and optimal thresholds calculated with the *pROC* package.^15^ To ensure robust validation, twofold cross-validation (CV) was employed, which involved repeatedly splitting the dataset into separate training and test sets with matched class sizes. This process was repeated 500 times, and mean AUCs for each metabolite were compared with randomly permuted data using the unpaired Student’s *t*-test. The reported AUC represents the mean AUC obtained from the full dataset across all CV runs.

All other analyses were performed using GraphPad Prism 10. The unpaired Student’s *t*-test with Sidak’s *post-hoc* testing was used where appropriate. Results were considered significant at p<0.05. Quantitative data are expressed as Tukey boxplots showing the IQR, with whiskers extending to minimum and maximum values within 1.5 x the IQR.

## Results

### Patient characteristics

The study cohort consists of 75 participants divided into non-progressors (n=52) and progressors (n=23) based on ΔEDSS per year. The groups were balanced for sex and age at the first sample. Consistent with the known female preponderance of MS,^16^ females constituted 82.2% of non-progressors and 70.0% of progressors. Disease-type distribution showed 73.0% RRMS and 13.5% SPMS in non-progressors, compared with 52.2% RRMS and 30.4% SPMS in progressors (p=0.16). Mean disease duration was similar between groups (11.5 vs 12.5 years; p=0.56), with a trend towards higher EDSS in progressors (3.6 vs 2.8; p=0.08). Medication usage varied, but no significant differences were observed (p=0.32).

### Increased TSPO ligand binding predicts progression

**Figure 1:**
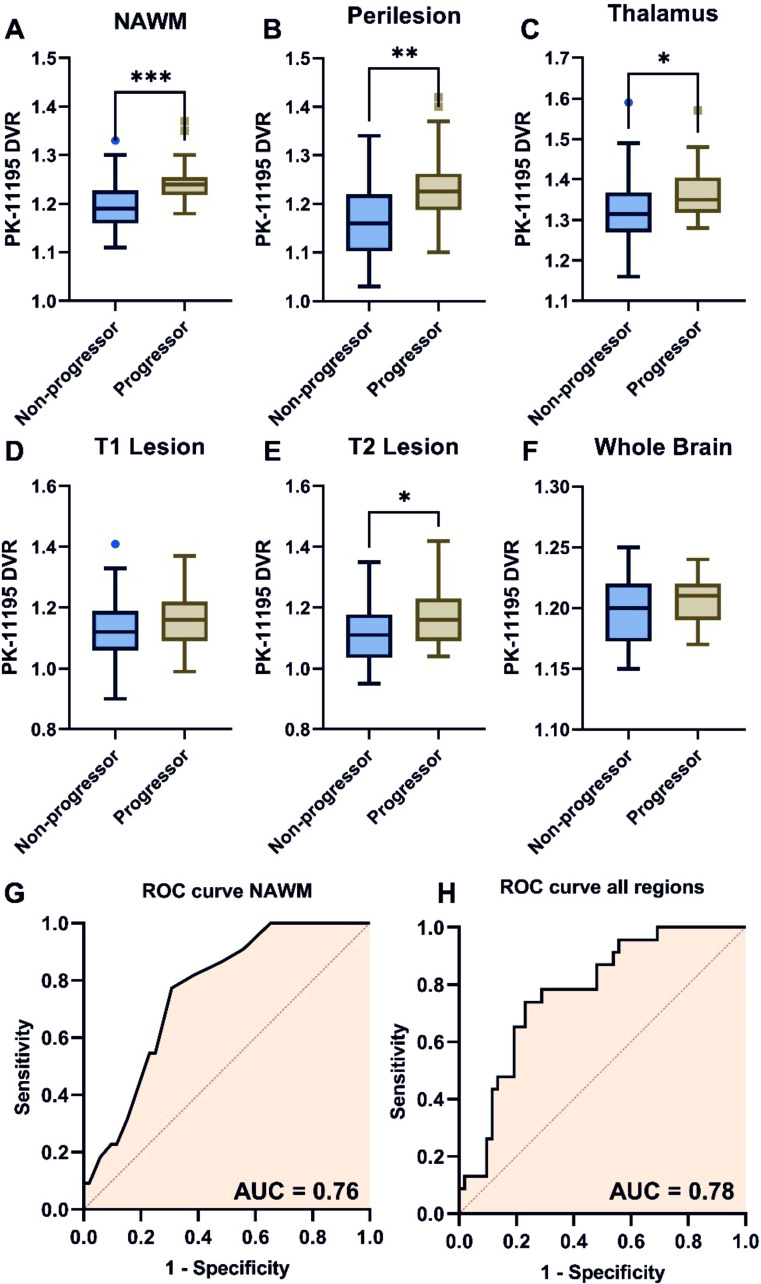
Extralesional ^11^C-PK11195 DVR is higher in multiple sclerosis patients with increased EDSS scores. DVR values for (**A**) the NAWM, (**B**) perilesional tissue, (**C**) thalamus and (**E**) T2 lesions were greater in progressors (ΔEDSS within 1 year of sampling, n=23), compared with non-progressors (n=52). No differences were observed in (**D**) the T1 lesion or (**F**) whole brain. (**G**) ROC curve analysis showed that NAWM DVR could distinguish between progressors and non-progressors, similarly to (**H**) the multi-region model. *p<0.05, **p<0.01 ***p<0.001. DVR, distribution volume ratio; EDSS, expanded disability status scale; NAWM, normal-appearing white matter.

To evaluate the relationship between PET imaging and NMR serum metabolomics, we first confirmed our previous finding that MS patients who progress exhibit increased ^11^C-PK11195 binding in the brain, indicating elevated brain inflammation in the normal appearing white matter (NAWM, figure 1A, p=0.0004), perilesional tissue (figure 1B, p=0.0018), and thalamus (figure 1C, p=0.04). ^1 11^C-PK11195 binding was significantly increased in MS patients who progressed within 1 year after sampling, compared with stable individuals, consistent with previous reports.^1 3^ No significant differences in ^11^C-PK11195 binding were observed between progressors and non-progressors within T1 lesions (figure 1D, p=0.08), despite significant alterations in T2 lesions (figure 1E, p=0.03), or in the whole brain (figure 1F, p=0.10). ROC curve analysis demonstrated the predictive utility of NAWM DVR in identifying progressors (figure 1G, AUC 0.76, p=0.0004). A multi-brain region logistic regression model produced a similar ROC curve (figure 1H, AUC 0.78, p=0.0002).

### Serum metabolome is associated with TSPO-defined brain inflammation

**Figure 2:**
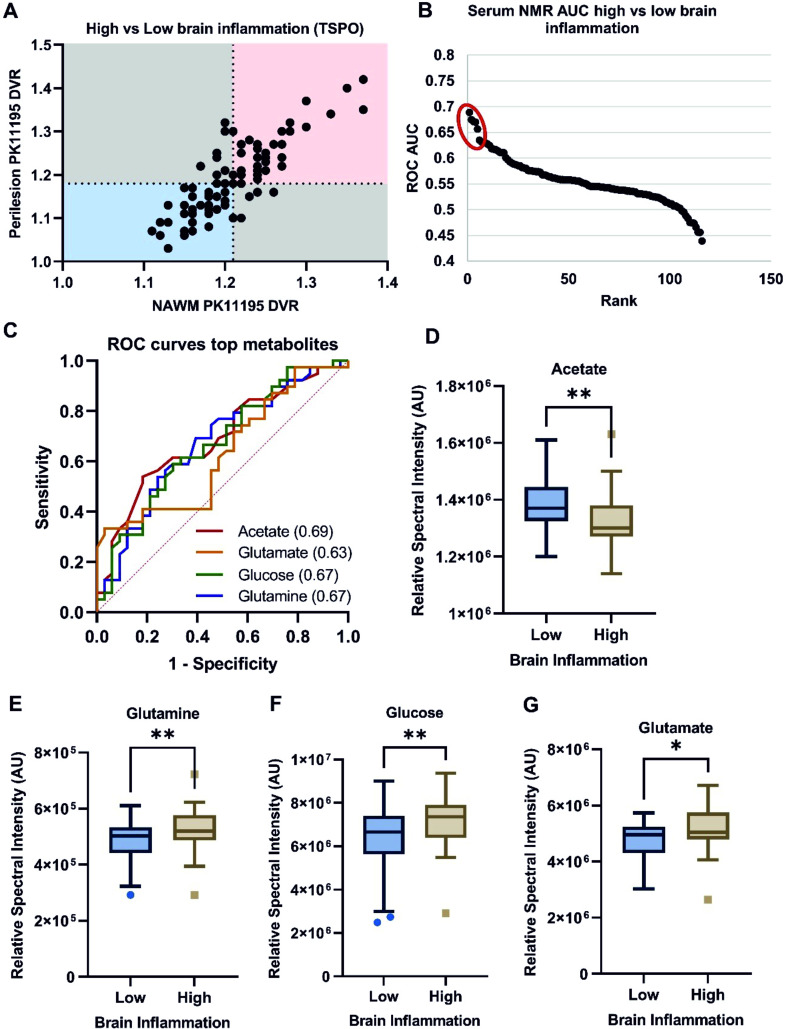
Selected serum metabolites vary with brain inflammation levels classified by ^11^C-PK11195 DVR. (**A**) Patients with high (n=39) and low (n=33) brain inflammation were classified by plotting NAWM DVR against perilesional DVR. Serum samples were measured by NMR and analysed using ROC curves and Student’s *t*-test. (**B and C**) Acetate, glutamate, glucose and glutamine showed moderate discriminatory capacity between high and low brain inflammation. (D) Acetate levels were lower in high inflammation (p=0.0099), while (E) glutamine (p=0.0064), (F) glucose (p=0.0076), and (G) glutamate (p=0.024) levels were increased. *p<0.05, **p<0.01, ****p<0.0001. DVR, distribution volume ratio; NAWM, normal-appearing white matter; NMR, nuclear magnetic resonance.

We investigated whether the serum metabolome could distinguish between patients with high and low brain inflammation to reduce reliance on PET imaging. To dichotomise the cohort, the ^11^C-PK11195 DVR for the NAWM and perilesional tissue were plotted for each patient (figure 2A), showing a strong positive correlation (Pearson’s correlation, r=0.83, p<0.0001). The median NAWM and perilesional tissue DVR values were used to determine cut-offs for high (red) and low (blue) brain inflammation, excluding patients with discordant DVR values (grey). This indicates that high brain inflammation directly influences the serum metabolic profile.

Subsequently, serum metabolites associated with brain inflammation were analysed (figure 2B). ROC analysis identified acetate (AUC 0.69, p=0.006), glutamine (AUC 0.67, p=0.011), glucose (AUC 0.67, p=0.013) and glutamate (AUC 0.63, p=0.056) as having moderate discriminatory capacity (figure 2C). Serum acetate levels were significantly lower in individuals with high brain inflammation (figure 2D), while glutamine (figure 2E), glucose (figure 2F), and glutamate (figure 2G) levels were increased.

### Trio of serum metabolites predicts progression with accuracy comparable to PET imaging

**Figure 3:**
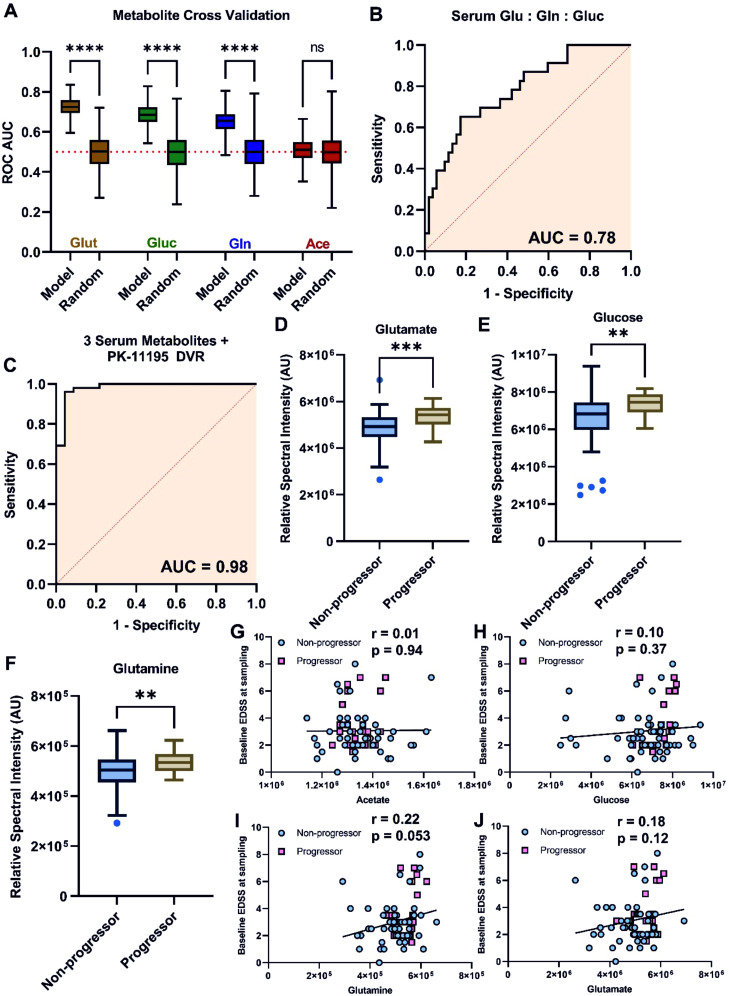
Serum glutamate, glucose and glutamine, but not acetate, independently predict disease progression. (**A**) Cross-validated accuracy of glutamate, glucose, glutamine and acetate, to predict progression, (**B**) combined metabolite model ROC AUC, (**C**) combined metabolite model ROC AUC with ^11^C-PK11195 DVR data. (**D**) Serum glutamate, (**E**) glucose, and (**F**) glutamine levels in progressors and non-progressors. Pearson correlations between baseline EDSS and serum (**G**) acetate, (**H**) glucose, (**I**) glutamine, and (**J**) glutamate. **p<0.01, ***p<0.001, ****p<0.0001. DVR, distribution volume ratio; EDSS, expanded disability status scale.

The magnitude of ^11^C-PK11195 binding is associated with progression, but it was unclear whether the serum metabolome, modestly linked to TSPO-defined brain inflammation, can predict MS progression. We investigated whether serum metabolites could predict progression (ΔEDSS) in MS. Glutamate, glutamine, and glucose (all p<0.0001), but not acetate (p=0.094), predicted progression within a year, with mean twofold cross-validated AUCs of 0.73, 0.65, 0.69, and 0.51, respectively (figure 3A).

Multiple logistic regression of serum glucose, glutamine, and glutamate levels discriminated progressors from non-progressors with an AUC of 0.78 (figure 3B, p=0.0001), correctly classifying 90.4% (47/52) of non-progressors and 43.5% (10/23) of progressors. Adding ^11^C-PK11195 DVR scores increased specificity, yielding an AUC of 0.98 (figure 3C, p<0.0001), correctly classifying 98.1% (51/52) of non-progressors and 87.0% (20/23) of progressors. Univariate analysis showed significant increases in serum glutamate (figure 3D, p=0.0006), glucose (figure 3E, p=0.0021), and glutamine (figure 3F, p=0.0052) in progressors.

Subsequently, EDSS at the time of sampling was plotted against absolute abundances of acetate (figure 3G), glucose (figure 3H), glutamine (figure 3I), and glutamate (figure 3J). Pearson’s correlation coefficient confirmed that metabolite abundances were independent of EDSS at the time of sampling.

### Predictive capacity of serum metabolites was validated in an independent MS cohort

**Figure 4:**
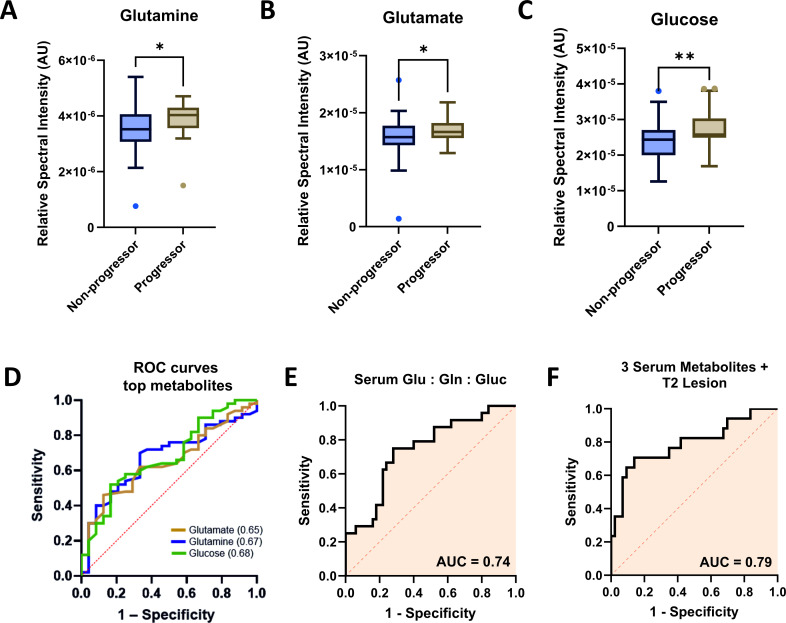
Serum metabolites predict disease progression in an independent cohort (SMSC). Serum (**A**) glutamine, (**B**) glutamate, and (**C**) glucose levels in progressors (n=12) and non-progressors (n=25). (**D**) ROC AUC of logistic regression for individual metabolites: glutamate, glutamine, and glucose. (**E**) Combined metabolite model ROC AUC, (**F**) combined metabolite model ROC AUC including MRI data - T2 lesion number. *p<0.05, **p<0.01.

To assess the reproducibility and generalisability of our findings, we performed an external validation using an independent cohort, the SMSC (online supplemental SI eTable 1). We validated the predictive capabilities of three serum metabolites, glutamine, glutamate, and glucose, demonstrating that all are significantly associated with increasing EDSS scores (figure 4A–C).

Univariate analysis in the SMSC cohort showed similar trends, with increased levels of glutamine (figure 4A, p=0.0377), glutamate (figure 4B, p=0.0409), and glucose (figure 4C, p=0.0027) in progressing MS individuals. ROC analysis confirmed the link between MS progression and serum metabolite levels, yielding AUC values of 0.67 for glutamine (p=0.016), 0.68 for glucose (p=0.014) and 0.65 for glutamate (p=0.037), indicating moderate discriminatory ability (figure 4D). Multiple logistic regression combining these metabolites produced an ROC AUC of 0.74 (figure 4E, p=0.0008), consistent with our previous model.

To further refine the model, we integrated radiological parameters, such as T2W lesion number, with NMR metabolites. This combined model yielded an ROC AUC of 0.79 (figure 4F, p=0.0005), demonstrating moderate improvement. These findings underscore the potential of the identified metabolites in predicting MS progression.

## Discussion

The magnitude of TSPO binding, reflecting CNS inflammatory activity, is elevated in MS patients and serves as a reliable predictor of disease progression.^1 3^ Here, we show that the serum metabolome, specifically, the levels of glutamate, glutamine, and glucose, are predictive of progression within 1 year later. By integrating the positive predictive power of TSPO ligand binding with the negative predictive power of metabolomics, we could achieve an exceptionally accurate (>95%) predictive model for disease progression. Moreover, our study demonstrates that NMR metabolomics offers a non-invasive, low-cost complement to TSPO-PET imaging for both short- and long-term monitoring of disease progression. The biomarker changes captured in our model not only mirror clinically relevant shifts in disability but also align with patient-reported outcomes, suggesting their potential to inform individualised treatment strategies. This integrative approach ultimately enhances patient management by delivering timely and accurate prognostic information.

### Elevated TSPO binding is associated with MS progression

Molecular imaging with TSPO PET ligands offers a valuable complement to MRI, revealing microglial and astrocyte activation not only in MRI-visible lesions but also in NAWM and normal-appearing grey matter (NAGM), providing a more comprehensive measure of disease burden.^17^ Our study shows higher TSPO binding in individuals with MS who progressed, compared with those who remained stable.^3^ In patients with increased EDSS within 1 year, the DVR values for NAWM, perilesional tissue, thalamic lesions and T2 lesions were elevated, while no differences were observed in T1 lesions or the whole brain, indicating non-homogeneous diffuse pathology.

### Serum metabolome as a surrogate marker of TSPO binding

Despite the predictive accuracy of TSPO-PET, its clinical translatability is constrained by the significant cost of radioligands and the limited availability of PET tracers. This creates an urgent need for more accessible and cost-effective blood-borne biomarkers. By using the well-established association between TSPO binding and MS progression as a ‘gold standard’, we aimed to identify alternative biomarkers that could address this need. Identifying such biomarkers has the potential to facilitate early treatment selection for silent progression, a critical gap in MS therapy, and could significantly enhance patient management and treatment outcomes. We, and others, have consistently shown that CNS inflammation and pathology can produce distinct metabolite signatures in the periphery.^18^,^20^ Here, we demonstrate that three serum metabolites—glutamine, glutamate, and glucose—significantly differ between patients with high and low TSPO binding and/or EDSS scores annual change, suggesting the serum metabolome can serve as a proxy for disease progression. Given the ease of blood sampling, the serum metabolome is a practical and cost-effective alternative to PET imaging. To our knowledge, this is the first study to directly compare peripheral metabolomics with CNS-PET imaging, highlighting its potential as a valuable tool in disease monitoring.

### Serum metabolome is predictive of progression

Cross-validated ROC analysis was used to evaluate the biomarkers initially identified as surrogates for TSPO binding in predicting disease progression. Glutamate, glucose, and glutamine were identified as highly predictive markers of disease progression, which was confirmed in an independent cohort. Glutamate is synthesised within the brain and is tightly regulated due to its potential neurotoxicity, with active transport from the brain to plasma to prevent excessive accumulation.^21^ Most amino acids have at least tenfold lower concentrations in brain extracellular fluid and cerebrospinal fluid compared with plasma, except for glutamine, for which the concentration is conserved on both sides of the blood–brain barrier.^22^ This is due to the role of glutamine as a precursor for neurotransmitters and its involvement in the glutamate–glutamine cycle, shuttling glutamate between neurons and astrocytes.^23^ Here, we speculate that increased blood glutamate may indicate neurotoxic export from damaged neurons or impaired uptake by activated astrocytes. Furthermore, activated microglia have also been shown to release glutamate in response to pro-inflammatory stimuli, necessitating increased glutamate efflux from the brain into the bloodstream.^24^ The increased serum glutamate levels thus may not only reflect ongoing neuroinflammation but also underscore the associated risk of neurotoxicity, particularly in individuals experiencing disease progression.

However, it is important to note that alterations in blood glutamate levels may not necessarily correspond to changes in brain glutamate. Instead, these levels could be influenced by peripheral pathophysiological processes in MS, such as leucocyte proliferation.^25^ We have previously demonstrated that changes in the blood metabolome of MS patients are associated with hepatic acute phase protein production and neutrophil recruitment to the liver.^26^ Furthermore, MS pathology extends beyond the brain, with significant effect on the gut–liver–brain axis signalling,^27^ suggesting that other organs affected by MS contribute to the blood metabolome.

### Use of ^11^C-PK11195

The utility of the TSPO-binding radioligand ^11^C-PK11195 for assessing brain-compartmentalised innate immune cell activity in MS has been well established over the past 25 years.^28^ Consistent with findings from seminal neuropathological studies,^29 30^ *in vivo* TSPO-imaging has revealed increased activation of innate immune cells in NAWM and chronic active lesions, with significant associations with progressive MS, higher EDSS and an increased risk of later disease progression.^31^ Importantly, the majority of TSPO-PET imaging studies in the context of MS have used the ^11^C-PK11195 radioligand.^3 32^ While the signal-to-noise ratio for ^11^C-PK11195 is somewhat limited due to poor brain penetration and high nonspecific binding,^33^ robust quantitation methods allow reliable *in vivo* assessment of the innate immune cell activity in both cross-sectional and longitudinal settings.^10 11 32^ New second-generation TSPO-specific PET radiotracers may enable glial activation monitoring with improved signal-to-noise ratios.^17^ One promising example is ^11^C-ER176, which offers enhanced signal-to-noise performance.^34^ However, no studies have yet explored the use of this radioligand in MS.

### Potential confounds

We confirmed that our results were not confounded by DMT use, as no significant differences in serum glutamine, glutamate and glucose levels were observed in DMT-treated individuals. This suggests that the serum metabolome reflects disease progression independently of DMT use. Evaluating DMT efficacy in preventing progression is challenging, requiring years of treatment to assess changes in disability, making early biomarkers for treatment response urgently needed. In this study, we were unable to demonstrate any DMT effect on either TSPO-PET signal (data not shown) or the serum metabolome, likely due to the variety of therapies (n=7), small treatment group sizes (n=2–13) and variable treatment durations, which are common consequences of retrospective studies. Previous cohorts have shown reduced TSPO-PET binding in MS patients on fingolimod^35^ and natalizumab,^32^ suggesting that DMT effects might be detectable in the serum metabolome.

## Conclusion

In summary, we identified that TSPO binding shows a strong correlation with key serum metabolites (glucose, glutamine, and glutamate) reflecting MS progression in two independent cohorts. Our findings underscore the pivotal role of TSPO binding as a marker in understanding disease dynamics. While NMR metabolomics offers valuable molecular insights and complements PET imaging, the strong association with TSPO binding highlights its potential for enhancing the accuracy of disease progression assessments. This synergy between TSPO imaging and metabolomics not only supports its feasibility for larger clinical trials but also promises to improve patient screening and monitoring for progressive MS treatment.

## Supplementary material

10.1136/bmjno-2025-001026 online supplemental file 1

## Data Availability

Data are available upon reasonable request.
