# Supplementary material for: Integrating TSPO-PET imaging with metabolomics for enhanced prognostic accuracy in multiple sclerosis
Source: BMJ Neurol Open. 2025 Apr 16;7(1):e001026. doi: 10.1136/bmjno-2025-001026 (PMC12004482; doi:10.1136/bmjno-2025-001026)
Supplement: online supplemental file 1 [file bmjno-7-1-s001.docx]

**Supplementary Information**

**Integrating TSPO-PET Imaging with Metabolomics for Enhanced Prognostic Accuracy in Multiple Sclerosis**

Daniel E. Radford-Smith^1^, Abi G. Yates^1,2^, Tereza Kacerova^2^, Marjo Nylund^3,4,5,6^, Marcus Sucksdorff^3,4,5,6^, Markus Matilainen^3,4,6^, Eline Willemse^,7,8,9,10^, Johanna Oechtering^7,8,9^, Aleksandra Maleska Maceski^7,8,9^, David Leppert^7,8,9^, Jens Kuhle^7,8,9^, Fay Probert^2^, Daniel C. Anthony^1^* & Laura Airas^3,4,5,6^

^1^Department of Pharmacology, University of Oxford, Mansfield Road, Oxford, UK

^2^Department of Chemistry, University of Oxford, Mansfield Road, Oxford, UK

^3^ Turku PET Centre, University of Turku, Turku University Hospital and Åbo Akademi University, Turku, Finland

^4^Clinical Neurosciences University of Turku, Turku, Finland

^5^Neurocenter, Turku University Hospital, Turku, Finland

^6^ InFLAMES Research Flagship, University of Turku, Finland

^7^Department of Neurology, University Hospital and University of Basel, Basel, Switzerland

^8^Multiple Sclerosis Centre, Departments of Biomedicine and Clinical Research, University Hospital and University of Basel, Basel, Switzerland

^9^Research Center for Clinical Neuroimmunology and Neuroscience Basel, University Hospital and University of Basel, Switzerland

^10^Department of Clinical Research, University Hospital and University of Basel, Basel, Switzerland

*Corresponding author

Professor Daniel C. Anthony

Department of Pharmacology

University of Oxford

Mansfield Road

Oxford

UK

Tel: +44(0)1865 281135

Email: daniel.anthony@pharm.ox.ac.uk

**eMethods**

**eMethods 1: Turku Cohort: Study Design and MS Patients**

The Turku Cohort MS patients were recruited from the outpatient clinic of the Division of Clinical Neurosciences at the University Hospital Turku, Turku, Finland between 2015 and 2019. The study protocol included ^11^C-PK11195 PET imaging to detect microglial activation, MRI to provide anatomical reference and to evaluate pathology related to MS, blood sampling to measure the concentration of serum metabolites, and clinical assessment by an experienced clinician to evaluate the EDSS score using standardised examination form (neurostatus.net). The Ethical Committee of the Hospital District of Southwest Finland approved the study and written informed consent was obtained from all participants according to the Declaration of Helsinki.

**eMethods 2: SMSC Cohort: Study Design and MS Patients**

The Swiss Multiple Sclerosis Cohort (SMSC; NCT02433028) is a prospective multicentre cohort study performed across eight Swiss academic medical centres (*1*). Demographic, neuroimaging, and clinical data as well as blood samples are collected every 6 or 12 months. Standardised clinical assessments with EDSS score calculations are performed by certified raters. Participants provided written informed consent. This study followed the Strengthening the Reporting of Observational Studies in Epidemiology reporting guidelines.

**eMethods 3: Turku Cohort: MRI acquisition and data analysis**

For the evaluation of multiple sclerosis pathology and for the acquisition of anatomic reference for the PET images, MRI with a 3 T Ingenuity TF PET/MR scanner (Philips) was performed. A semi-automated method was used first to create combined T2 lesion region of interest (ROI) mask image using the Lesion Segmentation Tool (LST, www.statistical-modelling.de/lst.html, a toolbox running in SPM8) and Carimas (https://turkupetcentre.fi/carimas/) for manual editing. A combined T1 lesion ROI mask image was manually shaped slice by slice, and the resulting T1 lesion ROI mask image was used to fill the corresponding T1 image with the lesion-filling tool in LST. The filled T1 was then used for segmenting grey matter, white matter and thalamus with the Freesurfer 5.3 software (http://surfer.nmr.mgh.harvard.edu/). The total T1 lesion load was measured from the manually edited T1 ROI masks. The individual T1 lesion core ROI masks were created by separation of individual lesions from the combined T1 lesion masks. Lesions ≤27 mm^3^ in size were excluded to avoid inclusion of unspecific T1 hypointensities.

**eMethods 4: SMSC Cohort: MRI acquisition and data analysis**

MRI assessment methods Brain MRI scans were performed annually in the SMSC. A standardised imaging protocol was applied across centers including a 3D Magnetization Prepared – Rapid Gradient Echo (MPRAGE), a 3D Fluid Attenuated Inversion Recovery (FLAIR) sequence, and a post contrast T1w sequence acquired at a spatial resolution of 1 mm^3^. T2 lesion volume (T2LV) was calculated automatically on FLAIR images using the multidimensional gated recurrent units algorithm, and results were manually reviewed by experts. Longitudinal changes of white matter lesions were automatically assessed with LeMan-PV, and the outputs, in terms of new and enlarged lesions (NEL), were manually reviewed and corrected. The number of CEL was assessed manually. T1w images were lesion-filled using the FSL-lesion filling tool10 and segmented by applying the SPM12 unified segmentation tool to compute gray matter (GMV), white matter (WMV) and CSF (CSFV) volumes. The total intracranial volume (TIV) was calculated as TIV = GMV + WMV + CSFV and total brain volume (TBV) as the sum of GMV and WMV.

**eMethods 5: Turku Cohort: Radioligand production and PET imaging acquisition**

The radiochemical synthesis of ^11^C-PK11195 was performed as previously described (*2*). The mean injected dose for was 476 ± 52 MBq [mean ± standard deviation (SD)] for the multiple sclerosis patient group. PET scan was performed using a brain dedicated ECAT HRRT scanner (CTI/Siemens) with an intrinsic spatial resolution of 2.5 mm. A 60 min dynamic PET scan was started simultaneously with an intravenous bolus injection of the ^11^C-PK11195 radioligand. Prior to the ligand infusion, a 6 min transmission scan for attenuation correction was obtained using a ^137^Cs point source. Thermoplastic head mask was used to minimize movement.

## **eMethods 6: Turku Cohort: PET post-processing and analysis**

PET images were reconstructed as previously described using 17 time frames. The reconstructed PET images were smoothed using a Gaussian 2.5 -mm post-reconstruction filter. Any displacement between frames were corrected using mutual information realignment in SPM8. Lastly, PET images were coregistered to T1 MRI and resampled to match the MRI voxel size 1 mm × 1 mm ×1 mm. The specific binding of ^11^C-PK11195 was evaluated using distribution volume ratio (DVR) in pre-specified ROIs. For the estimation of the ^11^C-PK11195 DVR, the time–activity curve corresponding to a reference region devoid of specific TSPO-binding was acquired for each PET session using a supervised cluster algorithm with four predefined kinetic tissue classes (SuperPK software). The reference tissue–input Logan method with a time interval from 20 to 60 min was applied to the regional time–activity curves using the supervised cluster algorithm grey reference input. For the individual lesion DVR analysis, the voxel-wise parametric binding potential (BPND) maps were calculated using basis function implementation of SRTM14 with 250 basis functions. Lower and upper bounds for theta were set to 0.06 and 0.8 1/min. The resulting parametric maps were normalized to Montreal Neurological Institute (MNI) space (MNI database) in SPM8 and the BPND images were transformed to DVR (DVR = BPND + 1).

**eMethods 7: NMR data processing**

All NMR spectra were phased, baseline corrected, and chemical shifts referenced to the lactate-CH_3_ doublet resonance at δ=1.33 ppm in Topspin 4.0.7 (Bruker). Spectra were then uploaded to ACD/NMR processor academic edition 12.01 (Advanced Chemistry development, Inc.) and regions were manually binned into ‘buckets’ corresponding to individual metabolites. The absolute value of integral of each spectral bucket was then retrieved (*3,4*).

**eMethods 8: NMR metabolite assignment**

Metabolite assignments were determined by referencing literature values, data from the Human Metabolome Database (*5*), and employing two‐dimensional total correlation spectroscopy (TOCSY) experiments to enhance the resolution and identification of metabolite resonances. TOCSY was particularly effective in resolving overlapping signals and confirming spin connectivity by providing detailed information on proton–proton correlations, thereby enabling precise metabolite identification. A standard Bruker dipsi2esgpph pulse sequence was utilised to acquire 2D 1H TOCSY spectra, employing 32 scans with a 0.08 s mixing time. In cases of residual ambiguity, serum samples were spiked with metabolite standards to validate the tentative assignments.

The analysis incorporated NMR-detectable serum/plasma metabolites previously reported (*3,4,6*). Among the most abundant metabolites observed in serum NMR spectra were lipoproteins - including high-density lipoproteins (HDLs), low-density lipoproteins (LDLs), very low-density lipoproteins (VLDLs), and chylomicrons - as well as amino acids (e.g., alanine, arginine, glutamate, glutamine, histidine, lysine, phenylalanine, proline, threonine, tyrosine, and valine), *N*-acetyl glycoproteins (GlycA and GlycB), and small molecules such as 3-hydroxybutyrate, creatine, creatinine, acetoacetate, citrate, formate, glucose, lactate, myo-inositol, scyllo-inositol, and urea.

**Supplementary Information**

**SI eTable 1: Baseline characteristics of individuals included in the SMSC** (all results are presented as means and were compared using Fisher’s exact test for categorical variables and Wilcoxon test for continuous variables)

|  | Non-progressors (n=25) | Progressors (n=12) | p-value |
| --- | --- | --- | --- |
| Age (mean years) | 46.0 | 46.0 | 0.49 |
| Sex (% female) | 60.0 | 66.6 | 0.69 |
| Race/Ethnicity  %Caucasian  % Other |  |  | >0.99 |
|  | 100.0 | 100.0 |  |
|  | 0.0 | 0.0 |  |
| Disease type  % RRMS  % SPMS  % Other/unknown | 64.0  16.0  20.0 | 25.0  41.7  33.3 | 0.07 |
| EDSS baseline | 3.60 | 3.67 | 0.44 |
| Medication at sampling (%)  None  Dimethyl fumarate  Fingolimod  IFN-β  Natalizumab  Ocrelizumab  Rituximab | 16.0  8.0  36.0  16.0  24.0  0.0  0.0 | 33.3  0.0  8.3  16.7  0.0  16.7  25.0 | 0.007 |

**Supplementary Information References**

**1.** Meier S, Willemse EAJ, Schaedelin S, et al. Serum Glial Fibrillary Acidic Protein Compared With Neurofilament Light Chain as a Biomarker for Disease Progression in Multiple Sclerosis. *Jama Neurology.* 2023;80:287-297.

**2.** Rissanen E, Tuisku J, Rokka J, et al. In Vivo Detection of Diffuse Inflammation in Secondary Progressive Multiple Sclerosis Using PET Imaging and the Radioligand (1)(1)C-PK11195. *J Nucl Med.* 2014;55:939-944.

**3.** Yeo T, Probert F, Sealey M, et al. Objective Biomarkers for Clinical Relapse in Multiple Sclerosis: A Metabolomics Approach (vol 3, 240, 2021). *Brain Communications.* 2021;3.

**4.** Yeo T, Sealey M, Zhou Y, et al. A blood-based metabolomics test to distinguish relapsing-remitting and secondary progressive multiple sclerosis: addressing practical considerations for clinical application. *Sci Rep.* 2020;10:12381.

**5.** Wishart DS, Guo A, Oler E, et al. HMDB 5.0: the Human Metabolome Database for 2022. *Nucleic Acids Res.* 2022;50:D622-D631.

**6.** Tang H, Wang Y, Nicholson JK, Lindon JC. Use of relaxation-edited one-dimensional and two dimensional nuclear magnetic resonance spectroscopy to improve detection of small metabolites in blood plasma. *Anal Biochem.* 2004;325:260-272.
